# Improving resilience in human milk banking: workshop proceedings, 11th November, London UK

**DOI:** 10.1186/s12919-026-00390-4

**Published:** 2026-08-12

**Authors:** Natalie Shenker, Amina Hatia, Samantha Griffin, Gemma Chandler-Maini, Liz Bailie, Debbie Barnett, Emma Savage, Alice D’Souza, Carmel Dowling, Tania Gane, Susan Stroud, Amanda Wood, Gemma Holder, Helen Crawley, Vicky Sibson, Helen Gray, Naomi Fallon, Melissa Brackley, Helen Hinds, Helen Turner, Matt Hogan, Debbie Kemp, Aileen Kennedy, Marko Kerac, Rehana Meeajan, Hema Mistry, Liz O’Sullivan, Clare Patton, Marta Staff, Laura Zuber, Merran Thomson, Asmaa Shariff, Vikki Psaila, Amanda Green, Jo Watt, Gemma Partridge, Tina Crombie, Flic Webster, Simon Cameron, Simran Cheema, Amy Brown, Gillian Weaver, Lucy Easthope

**Affiliations:** 1 Department of Surgery and Cancer, Imperial College London, IRDB, Hammersmith Hospital, Du Cane Road, London, W12 0HN UK; 2 Human Milk Foundation, Victory Road, Berkhamsted, Hertfordshire, HP4 1DL UK; 3 Northern Ireland Milk Bank, South West Acute Hospital, Enniskillen, Co. Fermanagh Northern Ireland; 4 Milk Bank Scotland, Elizabeth University Hospital Maternity Unit, 1345 Govan Road, Glasgow, Queen UK; 5 Milk Bank at Chester, Countess of Chester Health Park, Moston Lodge, Valley Drive, Chester, UK; 6 Peter’s Milk Bank, St Peter’s Hospital, Guildford Road, Chertsey, Surrey UK; 7 Southampton Milk Bank, Princess Anne Hospital, Coxford Road, Southampton, Hants UK; 8 Thomas’s Milk Bank, Lambeth Palace Road, London, UK; 9 Oxford Human Milk Bank, Neonatal Unit, John Radcliffe Hospital, Headley Way, Headington Way, Oxon, UK; 10 UKAMB Chair, Birmingham Women’s and Children’s NHS Foundation Trust, Edgbaston, Birmingham, UK; 11 First Steps Nutrition Trust, The Food Exchange, New Covent Garden Market, Studio 3.04, FounderLondon, UK; 12 Lactation Consultants of Great Britain, 19 Ascott Close, Hull, HU4 6EQ UK; 13 Centre for Food Policy, City University of London, Northampton Square, London, UK; 14 Johns Ambulance, St. John House, 5 Broadfield Close, Sheffield, South Yorkshire UK; 15 Lindisfarne Centre, Northumberland County Council, Lindisfarne Road, Alnwick, Northumberland UK; 16 British Transport Police Authority, British Transport Police FHQ, 25 Camden Road, London, UK; 17 London Resilience Group, London Fire Brigade, 169, Union Street, London, UK; 18 Essex County Council, County Hall, County Hall Market Road , Chelmsford, UK; 19 School of Biological, Health and Sports Sciences, Technological University Dublin, City Campus, Grangegorman, Dublin, Ireland; 20 Department of Population Health, London School of Hygiene & Tropical Medicine, Keppel Street, London, UK; 21 Institute of Epidemiology and Health Care, University College London, 1-19 Torrington Place, London, UK; 22 Warwick Clinical Trials Unit, University of Warwick, Coventry, UK; 23 Central Quad, Techological University Dublin, Grangegorman, Ireland; 24 Department of Human Rights Law, Queens University Belfast, Belfast, UK; 25 University of Exeter, Northcote House, Streatham Campus, Exeter, UK; 26 Department of War Studies, Kings College London, Strand, London, UK; 27 Consultant Neonatologist, Hillingdon Hospitals NHS Foundation Trust, London, UK; 28 Institute for Global Food Security, Biological Sciences Building, Queens University Belfast, 19 Chlorine Gardens, Belfast, Northern Ireland; 29 School of Health and Social Care, Swansea University, Singleton Park, Swansea, Wales; 30 Institute of Hazard, Risk and Resilience, Durham University, Stockton Road, Durham, UK

**Keywords:** Food insecurity, Emergency planning, Infant feeding, Breastfeeding, Human milk banking, Infant nutrition, Infant formula

## Abstract

Human milk banks (HMBs) are an essential clinical service, providing safe, screened donor human milk (DHM) to clinically vulnerable infants, which can enable mothers to establish their own milk supply. While HMB processes share similarities with other medical products of human origin (e.g., blood, platelets), HMBs remain marginalised services with limited resilience to external or internal pressures. As demand for DHM increases, understanding how to mitigate these vulnerabilities is essential. In November 2022, a workshop organised by the Human Milk Foundation brought together UK milk bank leaders, academics, and planners. The workshop aimed to understand existing pressures faced by UK HMBs and create a network to support strategic, evidence-based responses. Emergent themes highlighted that HMBs are stretched, facilitated largely by goodwill. Most services are reliant on limited workforces with limitations in service continuity, training, and succession planning. COVID-19 exacerbated pressures, but led to HMBs cooperating more. Proposed responses included investment in staffing, training and IT resources, greater public awareness and inter-HMB cooperation, and a national HMB Risk Register. Service continuity needs to be urgently addressed through external multiagency engagement, operational support, and research prioritization (including qualitative, technological and implementation science), defining optimal service design and financial resourcing to meet nationally agreed needs. Without intervention, the UK milk bank network may be unable to respond to future significant pressures, including specialist infant feed or infant formula shortages. With appropriate investment, a comprehensive National Service can be created to provide equitable services underpinned by robust research and innovation.

## Key Messages

- As marginalised services facing increasing demand, human milk banks need to be resilient to internal and external shocks.
- A 2022 workshop aimed to create a network to understand pressures on and improve resilience for UK human milk banks.
- Attendees concluded urgent needs to address staffing, funding and operational support, specifically for training and IT resource, and a national Risk Register.
- Appropriate investment would rapidly lead to a National Milk Bank Service that could provide service equity across the UK.

## Introduction

The 2016 Lancet Series on Breastfeeding confirmed the multiple health consequences to infants and mothers, and broader economic impacts on societies, of a lack of access to maternal milk [1, 2]. Burgeoning laboratory research into the properties of human milk has revealed how maternal milk is personalised for each infant, but shortfalls in maternal milk are common. Increasing attention is turning to understanding optimal alternative options for infant feeding and lactation support when maternal milk is limited or entirely unavailable, particularly for vulnerable infants. Donor human milk (DHM) from non-profit human milk banks (HMBs) is recommended by the World Health Organization (WHO) to feed vulnerable, usually preterm, infants when initial supplementation is needed, or when breastfeeding is impossible as part of a package of lactation support and to protect infants from life-threatening complications [3]. HMBs operate in a similar manner as the blood transfusion service, supporting donors who have extra milk to their own infant’s needs through health screening and blood tests, collecting, heat-treating (pasteurising) and storing donated milk, and continuing to support ongoing donors [4]. Human milk protects infants from life-threatening complications including necrotising enterocolitis (NEC) and bronchopulmonary dysplasia [5–9], and the provision of DHM has the overall aim of supporting maternal lactation where possible [10, 11].

Although milk banking services used to be more widely available, no national funding or HMB strategy has existed in the UK for over 40 years. Consequently, DHM largely became rationed for only the most premature infants where the risk of developing gut complications is higher. However, recent research has shown marked positive impacts of DHM availability alongside high quality lactation support in broader populations, including late preterm, full-term infants on the postnatal ward, and situations where maternal health affects the ability to lactate [12, 13]. Parental psychological wellbeing improves [14], anxiety and depression scores normalize [15], and preliminary findings suggest over 65% of mothers go on to establish breastfeeding who may otherwise have stopped [16]. This intervention therefore has major potential for public health benefits that are beneficial for mothers and infants [2].

The primary aim of this workshop was to define the current challenges and opportunities in terms of service continuity, resilience, and emergency planning for UK milk banking. The secondary aims were to identify major challenges and opportunities for HMB development and growth, foster a collaborative network of emergency planners and sector-specific experts, identify key issues for service continuity and reslience, identify research priorities, and ultimately work towards embedding planning for infant feeding within current UK emergency response plans.

## Methods

To discuss challenges facing UK milk banking, a diverse group of sector experts spanning milk banking, infant feeding and emergency planning was assembled at a workshop entitled, “Emergency planning in UK human milk banking” at a central venue in London on the 11th November 2022. Experts were identified from the published literature, in addition to known sectoral leads in key areas of emergency planning and HMB operational and academic experts. The strategic aim was to bring together a network of actively engaged individuals with a proven track record of driving a research programme or operational engagement in a related area.

A series of scene setting talks opened the day, with an overview of milk banking’s history, current UK provision, and some of the key challenges faced in the provision of both the NHS and charitable services. The participants then broke into four brainstorming groups, three in person and one online. Discussions were minuted and key themes transcribed to mind mapping boards, before being presented at the end of each session. Each group consisted of milk bank staff members, emergency planners, and academics with a range of interests in the sector, including supply chains, operational management, microbiology, psychology, and infant feeding in emergencies. Third sector organizations consisting of lactation support services and expertise in infant and young child feeding in emergencies were also represented. Participants were invited on the basis of their expertise. However, consideration was given to equity, diversity and inclusion across cultural backgrounds, workplace settings, geography, and career stages. Representatives attended from all UK countries and Ireland. Attendees unable to attend were offered the chance to join virtually, and 10 participants created a virtual group.

After the workshop, the recorded sessions were analysed with key emergent priorities regarding areas of service vulnerability extracted and recorded on Microsoft Excel (Microsoft, CA, USA). Opportunities to strengthen services were explored, both in terms of practicalities and knowledge gaps requiring research. Writing then continued on a shared platform (Google Docs, Alphabet, USA) so that all participants were able to contribute to the final draft of the manuscript.

## Results

### Areas of service vulnerability

There was intense discussion about the pressures on milk bank staff for maintaining services. Many of the discussions included how worthwhile, satisfying, and meaningful working in a milk bank was. However, a range of negative emotions were expressed by milk bank staff, including frustration at their milk bank being underfunded, particularly for staffing, exhaustion at frequently working beyond paid hours to maintain basic DHM provision, and signs of burnout expressed through anger and frustration at not being heard and working for too long with limited support. Staff members at smaller milk banks frequently described working from home and feelings of never being able to ‘switch off’.

Several attendees were working at milk banks that, in recent years, had experience of being unable to support their usual hospitals for multiple reasons, which had been exacerbated by the specific issues that affected milk banks globally since the pandemic [17]. The main reasons for this were around staffing, with under-resourcing contributing to stretched staff hours. When one member of the team was unwell or away, the capacity of the milk bank to function was reduced or removed. Staff in NHS milk banks were sometimes reallocated to clinical or other duties and felt that the significance of their milk bank role was not understood. Staffing levels varied greatly across the country, as has been described elsewhere. Some milk banks had less than one full time equivalent member of staff. There was often no continuity planning by the hospital for staff retirement, leaving one milk bank manager to return after her retirement to prevent the service closing.

As a result of under-resourcing, equipment levels were often minimal, meaning milk banks could stop functioning if key equipment failed or needed servicing. Sometimes, services would also reduce output if the printer failed. External supply chain issues also introduced a service vulnerability, which had worsened since 2020. Three milk banks reported a lack of single use jugs and blood testing vials for donor serology screening. These vulnerabilities were exacerbated by the lack of a culture of sharing problems between UK milk banks, meaning other milk banks were unaware and therefore unable to offer support [18].

There was also a lack of communication between the milk bank and external stakeholders, including individual hospital neonatal units and the neonatal operational delivery network (ODN), as well as local breastfeeding support groups and the general public. Communication was particularly difficult for smaller milk banks, generally those producing less than 1000 L a year, because of staffing resource within the bank.

Most of the larger milk banks had access to a bespoke human milk tracking and tracing system, as recommended in the National Institute for Clinical Excellence (NICE) Clinical Guideline #93 on the operation of a human milk bank [19]. However, most milk banks only had either paper-based or spreadsheet tracking, resulting in challenges in the storage of such data, but also less likelihood of a future tracking need being successfully completed. The main reason for a lack of tracking was resource allocation to the milk bank service, with business cases made by milk bank teams often rejected or subject to years of delay. The absence of a shared, robust tracking system potentially excludes some families from donating or accepting DHM due to cultural or religious concerns around milk kinship [20].

Milk banks are also vulnerable to reductions in the volume of milk donated, either through fewer potential donors coming forward, or through reduced volumes being donated from each individual. Often milk banks were limited in the numbers of donors that could be recruited for a range of reasons, including staffing levels, storage capacity for donated milk, and financial constraints to meet the cost of donor recruitment. Much of the general population remains unaware of the need for human milk to be donated, unlike the generally high levels of awareness regarding blood and organ donation, and others. Much discussion in the groups centered on the balance between raising awareness from its current very low levels and having the staff at the milk bank to appropriately support milk donors through the process of screening, recruitment, donation and completing donations. For donors with additional vulnerabilities, including those donating after the death of an infant, not having that support in place can lead to adverse impacts of trying to donate milk. Similarly, the negative consequences on public perceptions would be exacerbated if women try to donate milk and are refused or receive no response.

As there are only 13 functional milk banks in the UK, the logistics of collecting milk donations from homes and hospitals and distributing milk to end users is often complex. In the last decade a relationship has built between milk banks and local networks of blood bike volunteers, who provide courier services to the NHS for free. Milk banks identified difficulties with logistics resulting from a lack of available volunteers, adverse weather conditions, and frequent deliveries to neonatal units as a result of a lack of storage space in hospitals, HMBs and donor homes. The Hearts Milk Bank had trained its own volunteers to provide cover, but there were concerns about the training that would be needed for this to be rolled out nationally, and the resources to manage a larger network of volunteers. There was also risk through relying on unpaid volunteers to fulfill such a key role.

### Wider perceptions of service need: role of the Tombstone imperative?

This concept describes the investment in service provision to offset the likely risk of a high number of deaths as a consequence of a lack of provision. The rationale for DHM availability has for decades focused on the prevention of NEC, and the avoidance of deaths as a result. In the UK, equitable DHM provision has been estimated to save 247 neonatal deaths annually [21]. However, discussions reflected that the potential to help support breastfeeding would lead to improved health over the life course for both the infant and mother, including psychological health. In the UK, this is particularly pertinent, as suicide remains the highest cause of maternal death in the perinatal period [22, 23], and the risk of postnatal depression is exacerbated by a failure to achieve breastfeeding goals [23, 24]. DHM provision has been linked to improved breastfeeding outcomes in the context of optimal lactation support, and thereby may contribute to reduced avoidable deaths beyond those avoided through NEC reduction.

### Steps towards improving resilience

#### Develop a risk register

An immediate action is for each NHS milk bank to develop its own Risk Register, working with their local emergency preparedness team. NHS milk bank services are often embedded within neonatal services, but by virtue of the unique nature of their operations should have separate risk registers and engagement with the NHS Emergency Preparedness, Resilience and Response (EPRR) Framework process [25]. This is a statutory obligation on individual Trusts, and yet UK milk banks appear to be completely outside of the EPRR framework. For the Hearts Milk Bank, embedded within a standalone non-profit service, a Risk Register has been central to operations and actively updated since January 2020. However, the charity could improve its collaboration with local resilience frameworks, including local council resilience forums and local authorities. Some local authorities provide free service continuity planning assistance. Voluntary sector services can also link directly into NHS services through regional Voluntary Organisation Liaison Groups. Inclusion in such structures, along with enhanced inter-milk bank communication and an understanding of the diverse risks to service continuity, would provide greater early warning both to avoid disruptions in supply, and to mitigate DHM shortages to the end user.

#### Expand public awareness

Expanded public awareness is necessary, identified as achievable through combined strategies across social and conventional media, as part of improved breastfeeding education for school-age children, antenatal courses and healthcare services. The latter will be key in the future expanded provision, encompassing other areas of hospital services (e.g., postnatal wards, pediatric departments, surgical units, etc.) and community services (enhanced lactation support services, health visiting, community midwifery, etc.). As well as adapted university level education, CPD-accredited online modules would be useful to achieve education at scale while convenient to the professional.

#### Improve service cohesion

The overall sense from discussions was the isolated nature of milk banking services. Milk banking does not feature as national critical infrastructure, despite lifesaving impacts analogous to other tissue banking and organ transplant services [26]. Nor is there a unified platform for discussion between milk banks, regularly arranged and attended meetings, training and accreditation pathways or regulatory oversight,

Much discussion focused on engagement with decision makers, including NHS leadership and politicians. For these discussions to be fully informed, knowledge gaps need to be filled and research volume is slowly increasing across the sector.

### Future research priorities

UKRI-funded research is ongoing as to the most effective way to implement HMB services alongside hospital- and community-based lactation support, aiming to help reverse the UK’s status as having some of the lowest breastfeeding rates in the world. Identified research themes that should be prioritized were as follows:

- Qualitative research: Many of the potential indications for DHM use are understudied, including in looked after children, those needing palliative care, after maternal death, after maternal cancer treatment or prophylactic mastectomy and where the mother is trying to establish her own milk supply. Furthermore, there is no available evidence for the impact of DHM availability after disasters.
- Service implementation: For services to become resilient, there needs to be an understanding of optimal models, mechanisms to optimize collaboration and information sharing between stock levels (including real-time stock levels), and options for integration into pre-existing service continuity structures. Furthermore, there is a need for assessments of health economic data, both in terms of service delivery costings and cost-effectiveness, an understanding of the acceptability of DHM by both the general population and healthcare providers, and feasibility of scaling of services.
- Operational innovation: Milk banking processes have not changed for several decades. There are opportunities to introduce technological support that would enhance operational resilience, including digital tracking and tracing systems, improved pasteurisation and processing techniques, and logistics planning.

## Discussion

As research informs the clinical community on the role of human milk feeding in normal development and protection from life-threatening complications, as well as the support of maternal milk feeding, the implementation and safeguarding of milk banking services needs strengthening before scale-up. The need for expanded services has been highlighted by recently published global guidance from the WHO on the care of small and sick infants, which recommended DHM should be available as the first line nutrition option in the absence of maternal milk for all preterm infants, including but not exclusive to the very low birthweight population for whom DHM has largely been restricted to for over 40 years. In early 2023, BAPM published the first recommendations for DHM use within hospitals in the UK. Studies from 2017 highlighted that DHM was only used in 23% of very low birth weight infants [27], and although services in some regions, notably Scotland, the Southwest and Southeast, have grown, there is still an outstanding need. A UK neonatal unit survey in 2022 found that 87% of neonatal units (56% response rate) expected their provision of DHM to expand or start within the next 2 years. To meet the needs of all families cared for in hospitals or at home, a significant scale up of UK services is needed. Modelling suggests that approximately 25,000 L/annum is needed to meet the needs of preterm infants born up to 34 weeks cared for in hospital (Staff et al., unpublished data).

There are regional exceptions. The Southwest Milk Bank has received NHS England funding to meet the needs of all infants born up to 34 weeks gestation in the Southwest Operational Delivery Network (ODN). The PeriPREM study, a series of 11 Quality Improvement (QI) initiatives seeking to improve neonatal outcomes, including access to DHM to support breastfeeding, has reported positively [28]. There is currently no route to additional funding through NHS England though for this QI success to be rolled out to the other ODNs nationally, with the exception of the devolved governments.

Current output from NHS-funded HMBs is estimated at less than 10,000 L DHM/annum; the Hearts Milk Bank, set up by NHS professionals as a charity in 2017 now has a capacity of 5,000 L DHM/annum with only 40% of provision being funded through cost recovery payments from neonatal services.

HMBs should also be considered through the lens of food security. During the recent formula shortages in the United States (US), local milk banks increased capacity four-fold to meet the need of vulnerable infants in hospital or those unable to be fed with alternative formulas, playing an important role in the response [29], albeit limited by capacity [30]. Currently the UK would lack HMB capacity to respond to similar formula shortages. The UK also lacks legislation to prevent commercialisation of the sector with multiple documented ethical and safety harms [31, 32]. There are also no national, regional or local strategic plans for infant and young child feeding in emergency or disaster scenarios in the UK, a gap which is inconsistent with WHO guidance on infant and young child feeding in emergencies (IYCFE) [33].

The UK is not immune to disasters and formula shortages. Although the risk of the latter has to date not been quantified, the growing complexity of infant formula production and supply chains leaves this vital resource vulnerable [30]. With growing threats including climate change, floods, wildfires and human conflict posing an increasing risk to multiple communities, plans for all vulnerable populations should be made. Milk banks could play a vital role in safeguarding child health and breastfeeding relationships in disaster situations, but in the UK the network of National Health Service (NHS) milk bank services currently lacks the funding, infrastructure and training to enable the innovation and scale-up capacity. National guidance in the UK on emergency planning and resilience has failed to mention infants or infant feeding, and research highlights that only 29 LRFs in England and Wales even briefly mention infants and feeding supplies in evacuation guidance [33, 34]. Milk banks are also susceptible to service interruptions through other external pressures, most recently exemplified globally through the COVID-19 pandemic [18], but also from local emergencies such as power outages, as well as the aforementioned regional and national threats.

## Conclusions

Themes emerging from this Workshop highlight that milk banking is a stressed and stretched service, facilitated largely by the goodwill of a very small group of individuals. Most services are reliant on one or two members of staff, with minimal or absent service continuity, support for resilience or succession planning. These pressures were exacerbated by the COVID-19 pandemic, with shortages of DHM increasing as a consequence in 2021 and 2022. Vulnerabilities in service continuity need to be urgently addressed through external engagement, operational support, and research prioritisation, including defining optimal service design, staffing and financial resourcing to meet nationally agreed needs. Without such a programme, based on the principles of equity, sustainability, and evidence, the UK NHS milk bank network will continue to lack the ability to respond to future internal and external stressors, including significantly increased demand or UK-based shortages in formula or other specialist infant feeds. With appropriate investment, UK milk banking could rapidly coalesce into a standardised National Service, providing equity of access, robust evidence-based delivery of services that aim to support infant and maternal health, personalised therapeutics, and a highly active programme of research and innovation.

## References

[1] Rollins NC, Bhandari N, Hajeebhoy N, Horton S, Lutter CK, Martines JC, et al. Why invest, and what it will take to improve breastfeeding practices. Lancet. 2016;387(10017):491–504. 26869576 10.1016/S0140-6736(15)01044-2

[2] Victora CG, Bahl R, Barros AJ, França GV, Horton S, Krasevec J, et al. Breastfeeding in the 21st century: epidemiology, mechanisms, and lifelong effect. Lancet. 2016;387(10017):475–90. 26869575 10.1016/S0140-6736(15)01024-7

[3] WHO. WHO recommendations for care of the preterm or low-birth-weight infant. Geneva: World Health Organisation; 2022. 36449655

[4] PATH. Strengthening human milk banking: a resource toolkit for establishing & integrating human milk bank programs- a global implementation framework. Version 2.0. Seattle, Washington, USA: PATH; 2019.

[5] Quigley M, Embleton ND, McGuire W. Formula versus donor breast milk for feeding preterm or low birth weight infants. Cochrane Database Syst Rev. 2018;6:CD002971. 29926476 10.1002/14651858.CD002971.pub4 PMC6513381

[6] Quigley M, McGuire W. Formula versus donor breast milk for feeding preterm or low birth weight infants. Cochrane Database Syst Rev. 2014;4:CD002971. 10.1002/14651858.CD002971.pub3 24752468

[7] Boyd CA, Quigley MA, Brocklehurst P. Donor breast milk versus infant formula for preterm infants: systematic review and meta-analysis. Arch Dis Child Fetal Neonatal Ed. 2007;92(3):F169–75. 16556615 10.1136/adc.2005.089490 PMC2675323

[8] Blesa M, Sullivan G, Anblagan D, Telford EJ, Quigley AJ, Sparrow SA, et al. Early breast milk exposure modifies brain connectivity in preterm infants. Neuroimage. 2019;184:431–9. 30240903 10.1016/j.neuroimage.2018.09.045

[9] Villamor-Martínez E, Pierro M, Cavallaro G, Mosca F, Kramer BW, Villamor E. Donor human milk protects against bronchopulmonary dysplasia: a systematic review and meta-analysis. Nutrients. 2018. 10.3390/nu10020238. 10.3390/nu10020238 PMC5852814 29461479

[10] Shenker N, Associations VCNoHMBa. Maintaining safety and service provision in human milk banking: a call to action in response to the COVID-19 pandemic. Lancet Child Adolesc Health. 2020;4(7):484–5. 32573440 10.1016/S2352-4642(20)30134-6 PMC7202855

[11] Tyebally Fang M, Chatzixiros E, Grummer-Strawn L, Engmann C, Israel-Ballard K, Mansen K, et al. Developing global guidance on human milk banking. Bull World Health Organ. 2021;99(12):892–900. 34866685 10.2471/BLT.21.286943 PMC8640695

[12] Mondkar J, Chawla D, Sachdeva RC, Manerkar S, Shanbhag S, Khan A, et al. Impact of mother-baby friendly initiative plus approach on improving human milk feeding for neonates in hospital: a quality improvement before-and-after uncontrolled study. Eur J Pediatr. 2022;181(1):107–16. 34216269 10.1007/s00431-021-04141-9

[13] Merjaneh N, Williams P, Inman S, Schumacher M, Ciurte A, Smotherman C, Alissa R, Hudak M. The impact on the exclusive breastfeeding rate at 6 months of life of introducing supplementary donor milk into the level 1 newborn nursery. J Perinatol. 2020;40(7):1109–14. 10.1038/s41372-020-0657-6 32231257

[14] Brown A, Shenker N. Receiving screened donor human milk for their infant supports parental wellbeing: a mixed-methods study. BMC Pregnancy Childbirth. 2022;22(1):455. 35641919 10.1186/s12884-022-04789-7 PMC9154035

[15] Brown A, Griffin S, Weaver G, Shenker N. Receiving screened donor human milk as part of a community-based lactation support programme reduces parental symptoms of anxiety and depression. Matern Child Nutr. 2024;20(4):e13686. 38898718 10.1111/mcn.13686 PMC11574670

[16] Bramer S, Boyle R, Weaver G, Shenker N. Use of donor human milk in nonhospitalized infants: an infant growth study. Matern Child Nutr. 2021. 10.1111/mcn.13128. 10.1111/mcn.13128 PMC7988867 33404169

[17] Shenker N, Staff M, Vickers A, Aprigio J, Tiwari S, Nangia S, et al. Maintaining human milk bank services throughout the COVID-19 pandemic: a global response. Matern Child Nutr. 2021. 10.1111/mcn.13131. 10.1111/mcn.13131 PMC7883204 33403779

[18] Shenker N, Hughes J, Barnett D, Weaver G. Response of UK milk banks to ensure the safety and supply of donor human milk in the COVID-19 pandemic and beyond. Infant. 2020;16(3):118–21.

[19] NICE. Donor milk banks: the operation of donor milk bank services 2010. Available from: http://www.nice.org.uk/guidance/cg93. 22319806

[20] Bouzenita A. Islamic Bioethics: Milk Banks. In Oxford Research Encyclopedia of Religion. Oxford: Oxford University Press; 2023.

[21] Mahon J, Claxton L, Wood H. Modelling the cost-effectiveness of human milk and breastfeeding in preterm infants in the United Kingdom. Health Econ Rev. 2016;6(1):54. 27909996 10.1186/s13561-016-0136-0 PMC5133212

[22] MBRRACE-UK. Maternal mortality 2020–2022. 2024. Available from: https://www.npeu.ox.ac.uk/mbrrace-uk/data-brief/maternal-mortality-2020-2022.

[23] De Backer K, Wilson CA, Dolman C, Vowles Z, Easter A. Rising rates of perinatal suicide. BMJ. 2023;381:e075414. 37217225 10.1136/bmj-2023-075414

[24] Brown A, Rance J, Bennett P. Understanding the relationship between breastfeeding and postnatal depression: the role of pain and physical difficulties. J Adv Nurs. 2016;72(2):273–82. 26494433 10.1111/jan.12832 PMC4738467

[25] NHS England. Emergency Preparedness, Resilience and Response (EPRR). 2024. Available from: https://www.england.nhs.uk/ourwork/eprr/.

[26] Statista. Total number of organ transplants in the United Kingdom (UK) from 2009/10 to 2021/22. 2022. Available from: https://www.statista.com/statistics/380148/total-number-of-organ-transplants-in-uk/.

[27] Battersby C, Marciano Alves Mousinho R, Longford N, Modi N, UK_Neonatal_Collaborative_Necrotising_(UKNC-NEC)_Study_Group. Use of pasteurised human donor milk across neonatal networks in England. Early Hum Dev. 2018;118:32–6. 29454186 10.1016/j.earlhumdev.2018.01.017

[28] Glover Williams A, Tuvey S, McBain H, Menzies N, Hedge S, Bates S, et al. Perinatal excellence to reduce injury in preterm birth (PERIPrem) through quality improvement. BMJ Open Qual. 2022. 10.1136/bmjoq-2022-001904. 10.1136/bmjoq-2022-001904 PMC9367190 35944934

[29] Jackson F, Obeng C. Perceptions of human milk banks as a response to the US infant formula shortage: a mixed methods study of US mothers. Women Health. 2022;2(3):218–30.

[30] Kalaitzandonakes M, Ellison B, Coppess J. Coping with the 2022 infant formula shortage. Prev Med Rep. 2022. 10.1016/j.pmedr.2023.102123. 10.1016/j.pmedr.2023.102123 PMC9926015 36798794

[31] Klotz D, Wesołowska A, Bertino E, Moro GE, Picaud JC, Gayà A, et al. The legislative framework of donor human milk and human milk banking in Europe. Matern Child Nutr. 2021. 10.1111/mcn.13310. 10.1111/mcn.13310 PMC8932705 34936203

[32] Shenker N, Linden J, Wang B, Mackenzie C, Hildebrandt AP, Spears J, et al. Comparison between the for-profit human milk industry and nonprofit human milk banking: time for regulation? Matern Child Nutr. 2024;20(1):e13570. 37830377 10.1111/mcn.13570 PMC10749996

[33] WBTi UK Steering Group and Core Group. World Breastfeeding Trends Initiative UK Report 2016. 2016.

[34] Wise PA. Infant feeding in an emergency: An oversight in United Kingdom emergency planning. J Contingencies Crisis Manag. 2023;31:311–9.

